# Metamorphic remodeling of morphology and the body cavity in *Phoronopsis harmeri* (Lophotrochozoa, Phoronida): the evolution of the phoronid body plan and life cycle

**DOI:** 10.1186/s12862-015-0504-0

**Published:** 2015-10-21

**Authors:** Elena N. Temereva, Vladimir V. Malakhov

**Affiliations:** Department of Invertebrate Zoology, Biological Faculty, Moscow State University, Leninskie Gory 1-12, Moscow, 119234 Russian Federation

**Keywords:** External morphology, Phoronida, Metamorphosis, Blastocoel, Coelom, Body plan, Evolution, Ontogeny, Phylogeny

## Abstract

**Background:**

Phoronids undergo a remarkable metamorphosis, in which some parts of the larval body are consumed by the juvenile and the body plan completely changes. According to the only previous hypothesis concerning the evolution of the phoronid body plan, a hypothetical ancestor of phoronids inhabited a U-shaped burrow in soft sediment, where it drew the anterior and posterior parts of the body together and eventually fused them. In the current study, we investigated the metamorphosis of *Phoronopsis harmeri* with light, electron, and laser confocal microscopy.

**Results:**

During metamorphosis, the larval hood is engulfed by the juvenile; the epidermis of the postroral ciliated band is squeezed from the tentacular epidermis and then engulfed; the larval telotroch undergoes cell death and disappears; and the juvenile body forms from the metasomal sack of the larva. The dorsal side of the larva becomes very short, whereas the ventral side becomes very long. The terminal portion of the juvenile body is the ampulla, which can repeatedly increase and decrease in diameter. This flexibility of the ampulla enables the juvenile to dig into the sediment. The large blastocoel of the larval collar gives rise to the lophophoral blood vessels of the juvenile. The dorsal blood vessel of the larva becomes the definitive median blood vessel. The juvenile inherits the larval protocoel, mesocoel, and metacoel. Late in metamorphosis, however, the protocoel loses its epithelial structure: the desmosomes between cells and the basal lamina under the cells disappear. This loss may reflect a reduction of the protocoel, which is a characteristic of some recent phoronids.

**Conclusions:**

Based on our investigation of *P. harmeri* metamorphosis, we hypothesize that the phoronid ancestor was worm-like animal that possessed preoral, tentacular, and trunk coeloms. It lived on the soft sediment and collected food with its tentacles. When threatened, this worm-like ancestor buried itself in the soft sediment by means of the ventral protrusion into which the loop of the intestine and the blood vessels were drawn. We propose that this behavior gave rise to the body plan of all recent phoronids. The evolution of phoronid life cycle seems having more in common with“intercalation” than “terminal addition” theories.

**Electronic supplementary material:**

The online version of this article (doi:10.1186/s12862-015-0504-0) contains supplementary material, which is available to authorized users.

## Background

Metamorphosis is a remarkable event in the life cycle of some metazoans. It occurs in animals with indirect development and characterized by converting a larva, with a particular morphology, into a juvenile, with a different and equally distinctive morphology. Investigation of metamorphosis has great significance for understanding of the bilaterian life cycle and usually is discussed in the light of two main theories about origin of the bilaterian larvae [1–6]. According to “intercalation” theories the larval stages (planktotrophic or lecithotrophic) have evolved as specializations from the ancestral, direct life cycle [2–4]. The opposing “terminal addition” theories propose that the ancestor was holopelagic and that the adult stage was added to the life cycle with the pelagic stage retained as a planktotrophic larva [7–11]. Although investigation of metamorphosis has such a great significance for evolutionary analysis, metamorphosis of some enigmatic animals is still poor studied. One of these enigmatic groups is phylum Phoronida, whose metamorphosis is very unusual and the quickest among all animals with biphasic life cycle [1, 12].

The Phoronida is a small group of marine invertebrates with a biphasic life cycle. Adult phoronids live in their own tube in hard or soft substrata as benthic animals [13]. Their body completely embedded into substrata and only the anterior part of the body is exposed into the water. This portion of the body bears the lophophore – a special part of the mesosome, which performs several main functions including the collecting of food particles, the brooding of embryos, and respiration [13, 14]. Most phoronid species have planktotrophic larvae, actinotrochs, which live in plankton for one or several months [15–17]. By the end of pelagic life, larvae acquire some specific characteristics and then undergo catastrophic metamorphosis, which leads to formation of the definitive body plan [18].

Adult phoronids have an unusual body plan: their mouth and anus are located very near each other, and the digestive tract is U-shaped. Thus, in adult phoronids, the dorsal side is very short, whereas the ventral side is very long. On the other hand, phoronid larvae have a more standard organization with dorsal and ventral sides of similar length. Currently, there is only one explanation for how the unusual body plan of adults appeared during phoronid evolution [19]. This explanation presumes that a hypothetical ancestor of phoronids inhabited a U-shaped burrow in soft sediment, where it drew the anterior and posterior parts of the body together and eventually fused them [19]. As a consequence of folding, the paired coelomic sacks situated along the ascending and descending portions of the gut contacted each other and fused, forming the lateral mesenteries along the line of contact. In adult phoronids, the trunk coelom is divided into four chambers by five mesenteries [13]. Three of the mesenteries are parts of the anal-oral mesentery, which is common for most Bilateria, whereas the two lateral mesenteries are peculiar to phoronids and are not known in other bilaterians except the closest phoronid relatives, the brachiopods, some of which have one or even two pairs of lateral mesenteries [20, 21].

Although phoronid metamorphosis has been investigated many times by different methods [8, 22–27], a detailed description of the remodeling of the external morphology and the coelomic system during metamorphosis is lacking. Such a description might help to answer the question “What did the phoronid ancestor look like?”

The objectives of this work are to describe the remodeling of the external morphology and to trace the fate of body cavities during metamorphosis in *Phoronopsis harmeri*. Based on these findings, we will propose a new hypothesis about the evolution of the phoronid body plan. We will also consider some phylogenetic implications of our results and speculate about evolution of the phoronid life cycle.

## Methods

### Animals

Competent larvae of *P. harmeri* Pixell, 1912 were collected with a planktonic net during November of 2011 in Vostok Bay, Sea of Japan. In the laboratory, larvae were kept at 0–1 °C until metamorphosis. Many larvae underwent metamorphosis shortly after capture. For this reason, it was easy to collect metamorphic animals and fix them at 1–3 min intervals up to the newly formed juvenile – about 40 min after the onset of metamorphosis. Then 1-, 2- 3- 4-h-old, as well as 1-, 2-, 3-, 4-, and 9-day-old animals were collected and fixed for future investigations (see below). More than 30 individuals of each stage are investigated by different methods.

### Light microscopy

Competent larvae, metamorphic stages, newly formed juveniles, and 4-day-old juveniles were photographed using a Panasonic DMC-TZ10 digital camera (Panasonic, Kadoma, Japan) mounted on a binocular light microscope. All of these stages were prepared for histology, scanning electron microscopy (SEM), transmission electron microscopy (TEM), cytochemistry, and confocal laser scanning microscopy (CLSM).

### Histology

For histology, a 4 % paraformaldehyde (PFA) solution in filtered sea water was used as a fixative. Competent larvae, metamorphic stages of *P. harmeri*, newly formed juveniles, and 3- and 9-day-old juveniles were incubated in PFA for 8–10 h, rinsed in distilled water, dehydrated in ethanol, and embedded in Paraplast Regular (Sigma). Cross sections (5 μm thick) made with a Leica rotary microtome (Leica RM 2125; Leica Microsystems GmbH, Wetzlar, Germany) were stained with Caracci hematoxylin. Sections were examined with a Zeiss Axioplan2 microscope and photographed with an AxioCam HRm camera.

### Electron microscopy

For SEM, fixed metamorphic stages of *P. harmeri* that had been dehydrated in ethanol followed by an acetone series were critical point dried and then sputter coated with platinum-palladium alloy. Specimens were examined with a Jeol JSM scanning electron microscope (JEOL Ltd., Tokyo, Japan).

For TEM, metamorphic stages, newly formed juveniles, and 4-day-old juveniles of *P. harmeri* were fixed at 4 °C in 2.5 % glutaraldehyde in 0.05 M cacodylate buffer containing 21 mg/ml NaCl and then postfixed in 2 % osmium tetroxide in the same buffer containing 23 mg/ml NaCl. Postfixation was followed by en bloc staining for 2 h in a 1 % solution of uranyl acetate in distilled water. Specimens were then dehydrated in ethanol followed by an acetone series and embedded in Spurr resin (Sigma Aldrich). Semithin and thin sections were cut with a Leica UC5 ultratome (Leica Microsystems GmbH, Wetzlar, Germany). Semithin sections were stained with methylene blue, observed with a Zeiss Axioplan2 microscope, and photographed with an AxioCam HRm camera (Carl Zeiss, Oberkoche, Germany). Thin sections were stained with lead citrate and then examined with a JEOL JEM 100B electron microscope (JEOL Ltd., Tokyo, Japan).

### Cytochemistry

For cytochemistry, metamorphic stages of *P. harmeri*, newly formed juveniles juveniles were narcotised in MgCl_2_, fixed overnight in a 4 % paraformaldehyde solution in filtered sea water, and washed two times in phosphate buffer (pH 7.4) (Fisher Scientific) with Triton X-100 (0.3 %) (Fisher Scientific, Pittsburgh, PA, USA) for a total of 2 h. Then, the specimens were washed in PBT (phosphate buffer + Triton) and incubated in a mixture of rhodamine-conjugated phalloidin (1:50) (Fisher Scientific, Pittsburgh, PA, USA) for 1 h at room temperature in the dark. They were subsequently washed in phosphate buffer (three times × 40 min), mounted on a cover glass covered with poly-L-lysine (Sigma-Aldrich, St. Louis, MO, USA), and embedded in Murray Clear. Specimens were viewed with a Leica TCS SP5 confocal microscope (IDB, Moscow, Russia). Z-projections were generated using the program Image J version 1.43.

### Ethics statement

The use of phoronids in the laboratory does not raise any ethical issues and therefore approval from regional or local research ethics committees is not required. The field studies did not involve endangered or protected species.

## Results

### Morphology of competent larvae

**Fig. 1:**
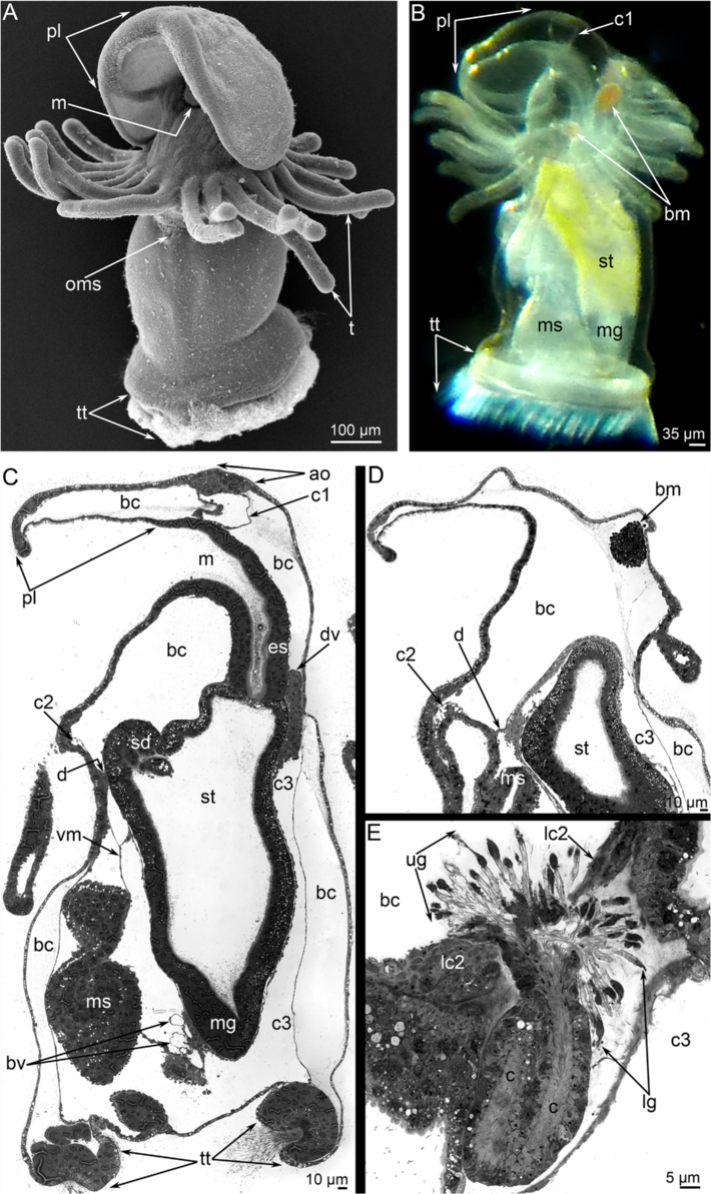
Organization of the competent larvae of *Phoronopsis harmeri.* In all photographs, the apical side is to the top. **a** Whole larva viewed from the ventro-lateral side; SEM. **b** Photograph of a live larva viewed from the left. **c** Sagittal semithin section of whole larva; the ventral side is to the left. **d** Longitudinal semithin section of the preoral lobe of larva; the ventral side is to the left. **e** Longitudinal semithin section of the protonephridium, which bears upper and lower groups of terminal cells. Abbreviations: ao – apical organ; bc – blastocoel; bm – blood mass; bv – blood vessels; c – canal of protonephridium; c1 – protocoel; c2 – mesocoel; c3 – metacoel; d – diaphragm; dv – dorsal blood vessel; es – esophagus; lc2 – mesocoel lining; lg – lower group of terminal cells; m – mouth; mg – midgut; ms – metasomal sac; oms – opening of metasomal sac; pl – preoral lobe; sd – stomach diverticulum; st – stomach; t – tentacle; tt – telotroch; ug – upper group of terminal cells, vm – ventral mesentery

**Fig. 2:**
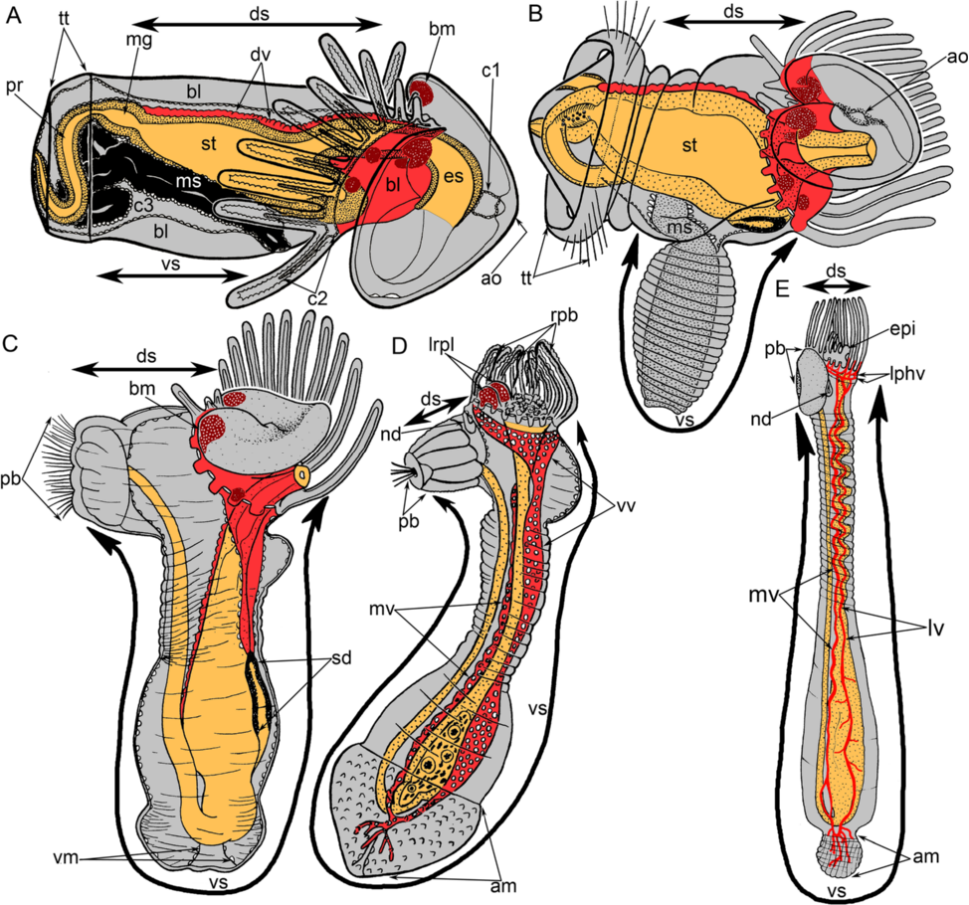
A scheme of consecutive stages of metamorphosis of *Phoronopsis harmeri*. In all panels, the dorsal side is to the top, the ventral side is to the down. **a** Competent larva. **b** Start of eversion of the metasomal sac. **c** The metasomal sac is completely everted. **d** Stage of eating of the preoral lobe and the postoral ciliated band. **e** 4-h-old juvenile with completely formed blood system. Abbreviations: am – ampulla; ao – apical organ; bc – blastocoel; bm – blood mass; c1 – protocoel; c2 – mesocoel; c3 – metacoel; ds – dorsal side of the body; dv – dorsal blood vessel; epi – epistome; es – esophagus; lphv – two lophophoral blood vessels; lrpl – two lateral remnants of preoral oobe; lv – ventrolateral blood vessel; mg – midgut; ms – metasomal sac; mv – median blood vessel; nd – nephridial duct; pb – posterior bulge; sd – stomach diverticulum; st – stomach; t – tentacle; tt – telotroch; vm – ventral mesentery; vs – ventral side of the body; vv – ventral blood vessel

Before metamorphosis, *P. harmeri* larvae are transparent, have 24 tentacles, and are 1400–1500 μm long (Fig. 1a, b). The competent *P. harmeri* larva has a large preoral lobe, a collar region with tentacles, and a trunk with a terminal telotroch that bears long cilia (Fig. 1a). Because the integument is transparent, some details of the internal organization of the larva can be readily observed. In the preoral lobe, the closed cylinder-like cavity is located between the apical plate and esophagus (Fig. 1b). This cylinder-like cavity is the preoral coelom (protocoel), which is also easily recognized in sagittal sections of the larva (Fig. 1c). The collar region is occupied by a large blastocoel, which contains several masses of red erythrocytes (Fig. 1b, d). The blastocoel is crossed by numerous thin fibers of extracellular matrix (ECM) (Fig. 1c, d). The mesocoel is small and is located at the tentacle base (Fig. 1d). The mesocoel contacts the peripheral part of the upper border of the metacoel, and together they form the diaphragm (Fig. 1c, d). A pair of protonefridia is located on the lateral sides of the trunk, under the tentacles. Each protonephridium consists of a U-shaped duct, which is located in the blastocoel between the body wall and metacoel, and two groups of terminal cells (Fig. 1e). The upper group is extends into the large blastocoel of the collar; the lower group is located in the blastocoel between the mesocoel and metacoel. The metacoel is the largest coelom in the larva (Fig. 1c). The internal portion of the upper border of the metacoel, which does not contact the mesocoel, is attached to the digestive tract under the stomach diverticulum. The lateral walls of the metacoel are separate from the body wall, and thus there is a spacious blastocoel, which surrounds the metacoel as a sac. The external walls of the metacoel are very thin, whereas the internal walls are much thicker, especially on the dorsal side of the stomach where the dorsal blood vessels are located (for details see [28]) (Fig. 1c). The stomach, which is yellow, is located in the upper portion of the trunk and continues to the transparent, funnel-like midgut (Fig. 1b, c). The white metasomal sac mostly passes along the ventral side of the trunk (Figs. 1b and 2a). The metasomal sac is the invagination of the epidermis on the ventral side under the tentacles, where the small opening of this invagination is visible (Fig. 1a). The metasomal sac is attached to the body wall and digestive tract by ventral mesentery (Fig. 1c).

### Metamorphosis

#### Remodeling of external morphology

**Fig. 3:**
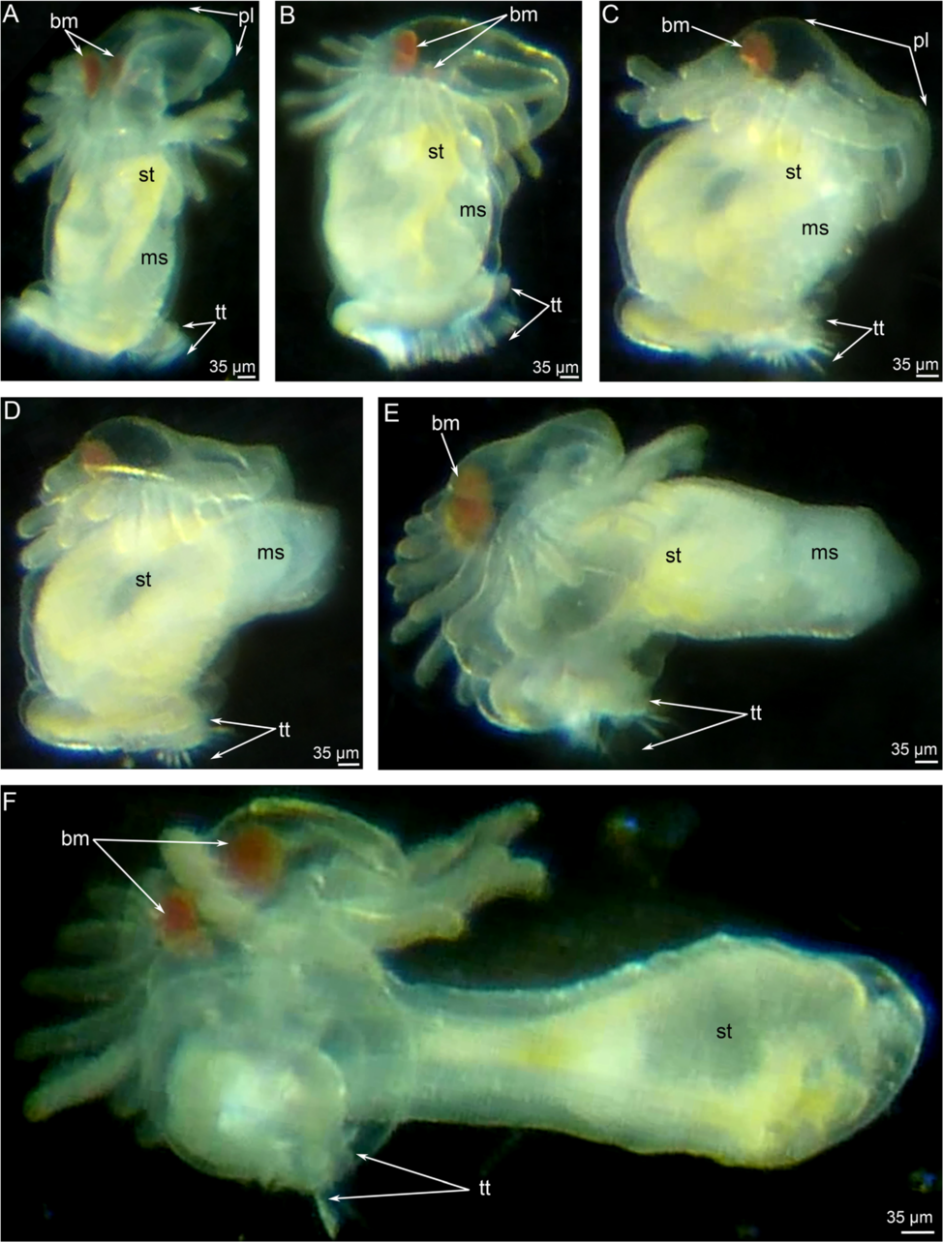
First steps of metamorphosis of *Phoronopsis harmeri*; photographs of live animals. In all photographs, the larval preoral lobe with apical organ is to the top. **a** Competent larva. **b** First contraction of larval muscles. **c** Strong contraction of larval muscles: larval length decreases in twice. **d** Start of eversion of the matasomal sac. **e** The metasomal sac is everted in half. **f** The metasomal sac is completely everted. Abbreviations: bm – blood mass; ms – metasomal sac; pl – preoral lobe; st – stomach; tt – telotroch

**Fig. 4:**
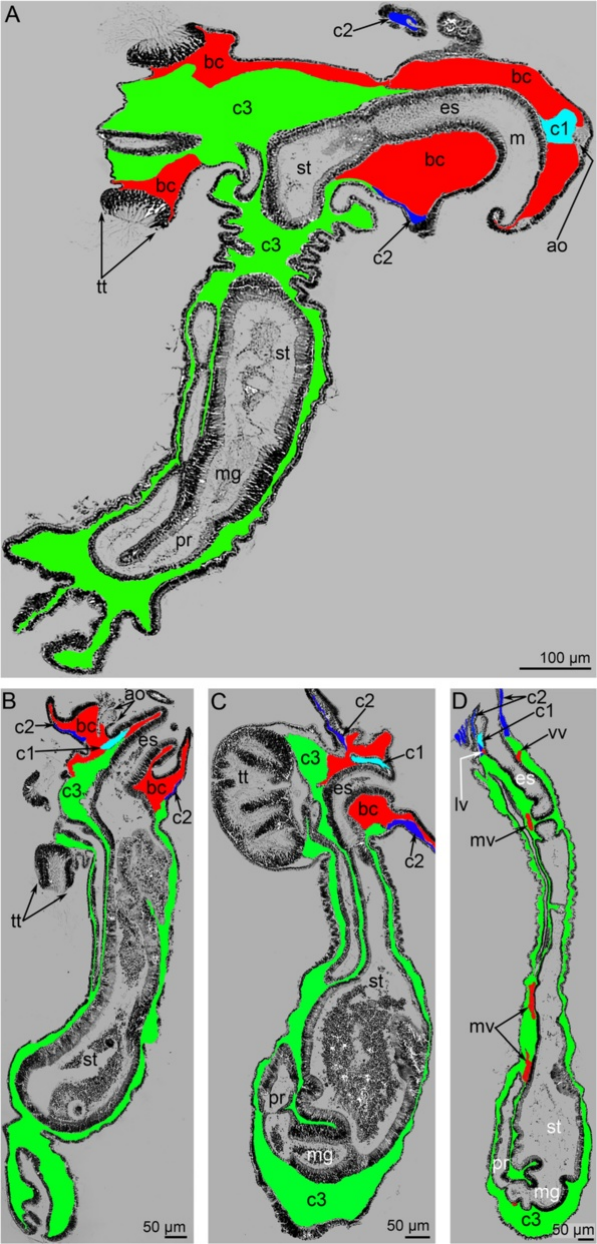
Remodeling of body cavities during metamorphosis of *Phoronopsis harmeri*. Histological sagittal sections of animals at consecutive stages of metamorphosis. Color code: red – blastocoel, which partly gives rise to the blood system; cyan – protocoel; blue – mesocoel; green – metacoel. **a** First step of metamorphosis: larva with everted metasomal sac. Stage about 1 min after the onset of metamorphosis. Larval apical organ is at the right; telotroch is at the left; metasomal sac is to the down. **b** Stage of eating of the preoral lobe; about 10 min after the onset of metamorphosis. Degenerated apical organ is well visible at this stage. **c** Stage of eating of the postoral ciliated band: stage about 12–15 min after the onset of metamorphosis. **d** 9-day-old juvenile completely acquired definitive body plan. Abbreviations: ao – apical organ; bc – blastocoel; c1 – protocoel; c2 – mesocoel; c3 – metacoel; es – esophagus; lv – lophophoral blood vessel; m – mouth; mg – midgut; mv – median blood vessel; pl – preoral lobe; pr – proctodaeum; st – stomach; tt – telotroch; vv – ventral vessel

**Fig. 5:**
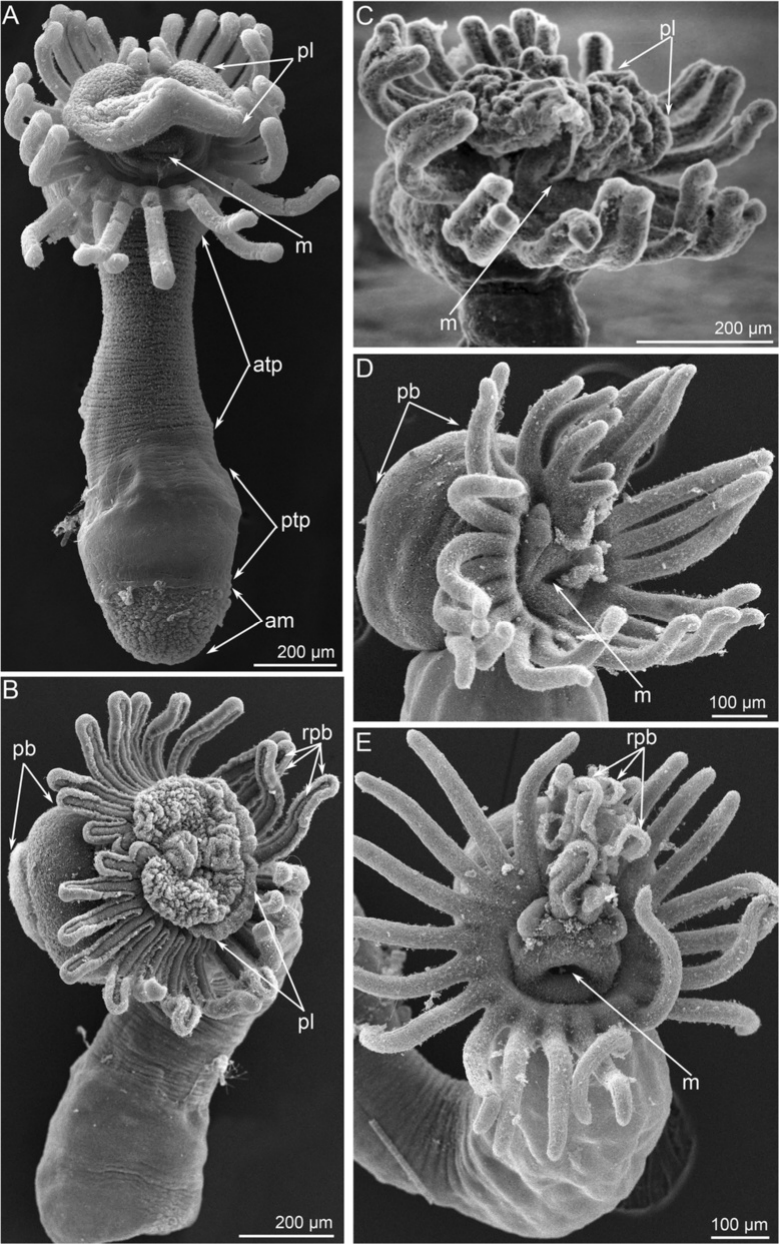
First steps of metamorphosis of *Phoronopsis harmeri* accordingly to SEM. In all photographs, apical is to the top. **a** Metamorphic animal with completely everted metasomal sac: stage about 1 min after the onset of metamorphosis. Difference in organization of the trunk epithelium allows to mark several zones: anterior trunk part, posterior trunk part, and ampulla. **b** Metamorphic animal with degenerated preoral lobe, huge posterior bulge, and continuous rope of postoral ciliated band. Stage about 10–12 min after the onset of metamorphosis. **c** Metamorphic animal is eating the preoral lobe. Macerated epithelium of the preoral lobe is involved into the mouth. **d** Metamorphic animal without the preoral lobe. **e** Metamorphic animal at stage of formation and eating of the continuous rope of postoral ciliated band. Abbreviations: am – ampulla; atp – anterior trunk part; m – mouth; pb – posterior bulge; pl – preoral lobe; ptp – posterior trunk part; rpb – rope of postoral ciliated band

**Fig. 6:**
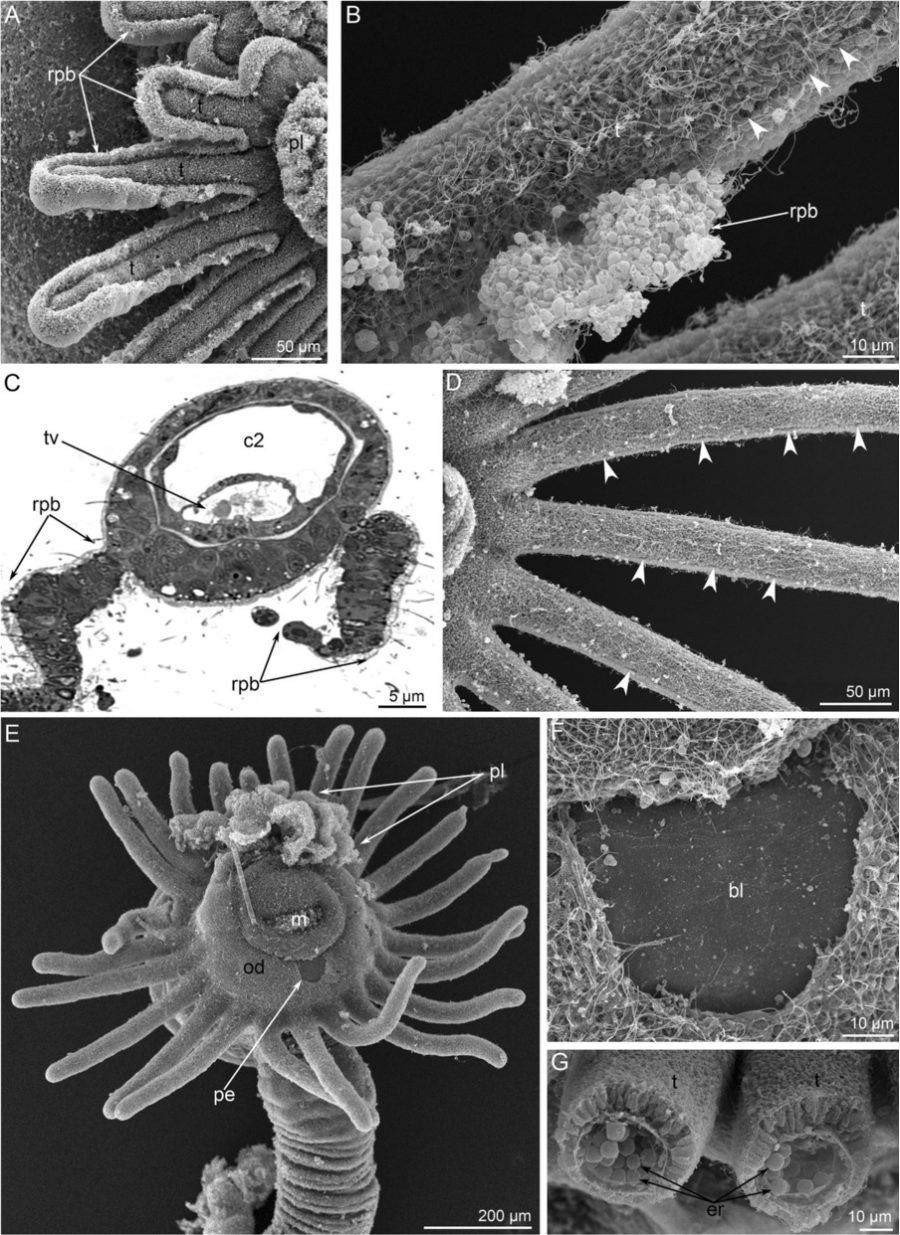
Details of metamorphic remodeling of external morphology in *Phoronopsis harmeri*. Photographs according to SEM (A-B, D-G) and semithin section (**c**). **a** Tentacles (t) of metamorphic animal with continuous rope of postoral ciliated band (rpb). **b** A portion of tentacle with degenerated epithelium of postoral ciliated band: line of former location of the postoral ciliated band is indicated by arrowheads. **c** Pair of latero-frontal ropes of postoral ciliated band is evident on each tentacle. **d** Tentacles after remodeling: line of former location of the postoral ciliated band is indicated by arrowheads. **e** Anterior portion of the body in metamorphic animal with partly consumed preoral lobe (pl) and spacious oral disc (od) with peeled epithelium (pe). **f** A portion of the oral disc is covered by basal lamina (bl). **g** Cross section of juvenile tentacles (t) with erythrocytes (er). Abbreviations: c2 – mesocoel; m – mouth; tv – tentacle vessel

**Fig. 7:**
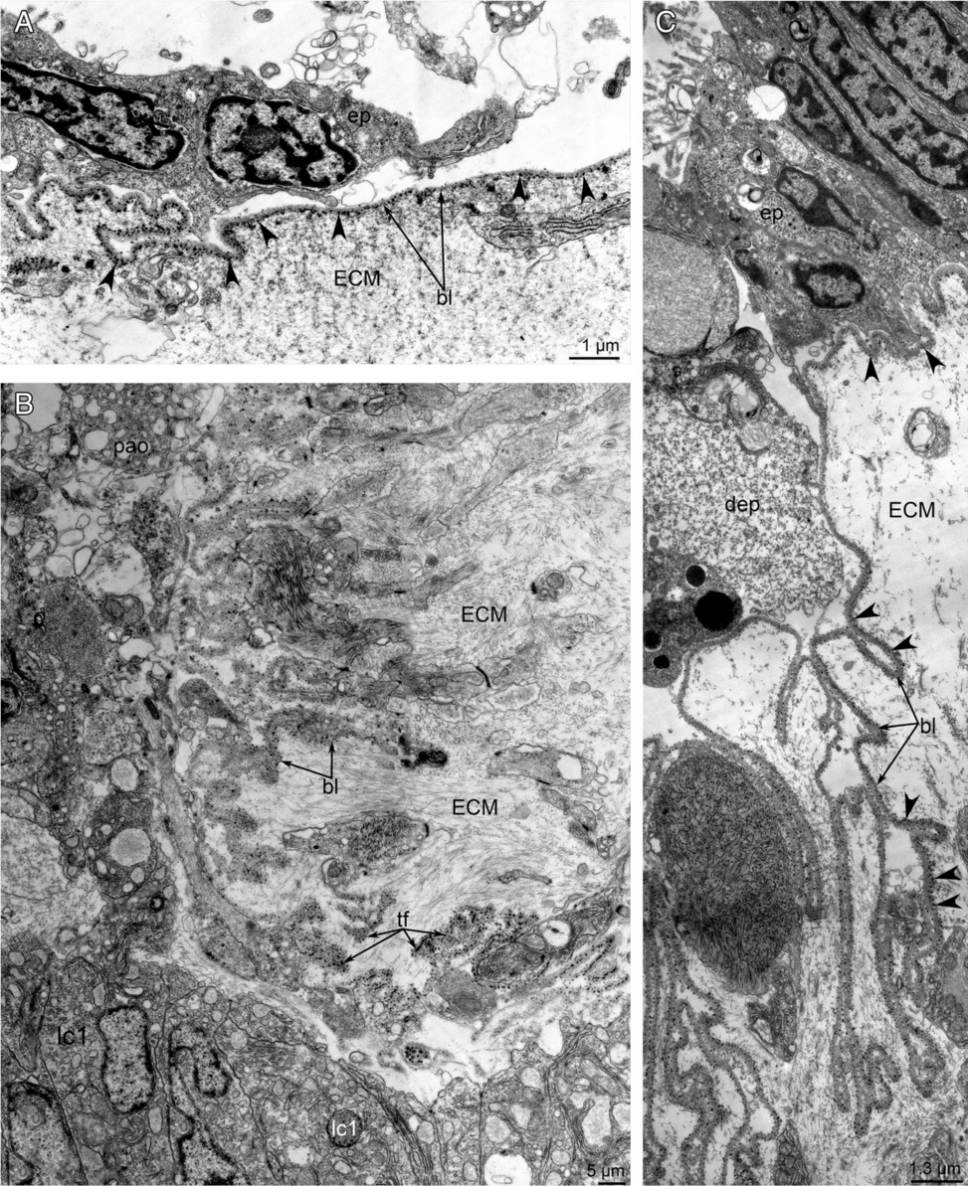
Ultrastructural remodeling of integument during metamorphosis of *Phoronopsis harmeri*. Stage about 15 min after the onset of metamorphosis. **a** Epithelium (ep) and basal lamina (bl) of the oral disc. Thick electron dense fibrils are indicated by arrowheads. **b** Thick extracellular matrix (ECM), numerous protrusions of the basal lamina, and degenerated muscle cells in the preoral lobe near the protocoel. **c** The basal lamina forms long protrusions, which penetrate profoundly into the ECM. Thick electron dense fibrils are indicated by arrowheads. Abbreviations: dep – degenerated epithelium; lc1 – lining of the protocoel; pao – pilled apical organ; tf – thick electron dense fibrils

**Fig. 8:**
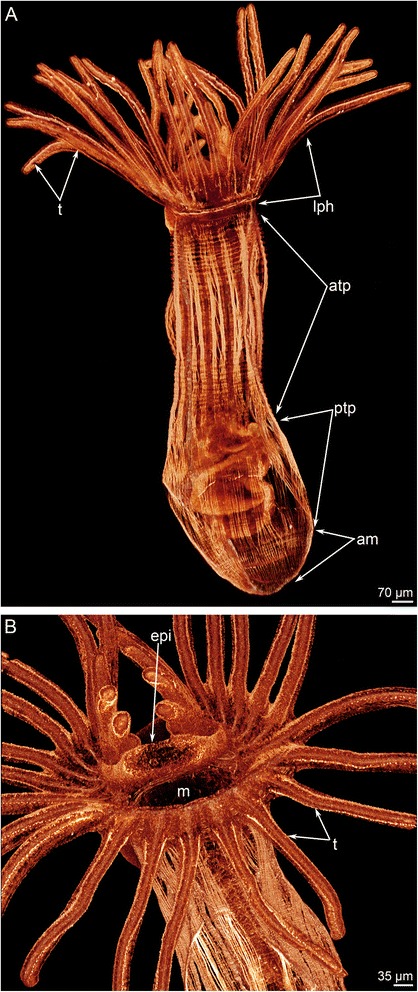
Newly formed juveniles of *Phoronopsis harmeri* stained for phalloidin. Stage about 40 min after the onset of metamorphosis. **a** Whole animal. According to specific organization of the muscles, juvenile body is subdivided into the lophophore (lph) with tentacles (t), anterior trunk part (atp), posterior trunk part (ptp), and ampulla (am). **b** Anterior part of the body: completely formed epistome (epi) is well visible above the mouth (m)

Metamorphosis begins with a great contraction of the larval muscles (for details see [26]), which produces high pressure in the trunk coelom and causes the eversion of the metasomal sac (Figs. 2b and 3a–f). The eversion requires about 5 min; the most distal part of the metasomal sac is extremely flexible and repeatedly increases and decreases in diameter (Additional file 1). Because the digestive tract connects to the metasomal sac via the ventral mesentery, it is drawn into the metasomal sac and acquires U-shaped structure (Figs. 2c, 3f and 4a). Together with digestive tract, the larval blood vessels are drawn into the metasomal sac. After eversion of the metasomal sac, the larva acquires a worm-like shape (Figs. 2c and 5a). The posterior portion of the larval body turns into a large bulge, which surrounds the larval telotroch (Fig. 4c and 5b, d). In the next stage of metamorphosis (stage about 10 min after the onset of metamorphosis), the preoral lobe and all of its parts (the apical and frontal organs) undergo cell death and then are consumed by the juvenile (Figs. 2d and 5b). Cell debris, which is readily observed with SEM, is drawn into the mouth and then consumed (Fig. 5c). Simultaneously, the epidermis of the postoral ciliated band degenerates: it forms a continuous rope-like structure, which passes along all tentacles and then is squeezed from the epidermis and consumed by the juvenile (Figs. 2d, 5d and 6a). Along the lateral sides of each juvenile tentacle, the thin line, which lacks of epidermis, extends (Fig. 6b, d). In the first stages of metamorphosis (stage about 12 min after the onset of metamorphosis), the extruded epithelium of the postoral ciliated band forms two ropes of cellular debris along the latero-frontal sides of each tentacle (Fig. 6c). In juveniles (stage about 40 min after the onset of metamorphosis), the tentacles are evenly covered by cilia and lack the specific ciliated zones that are characteristic of adult and larval tentacles (Fig. 6d). Often during metamorphosis, the oral disk is pushed forward, and the area around the mouth becomes visible (Figs. 5e and 6e). When this occurs, it makes it evident that the epidermis of the oral field has peeled, and the area, which is covered only by the basal lamina, appears near the mouth (Fig. 6e, f). According to TEM, this area is covered by a thick basal lamina, which contains many, thick, electro-dense fibrils and rests on amorphous ECM (Fig. 7a). The newly formed juvenile has a long body (stage about 40 min after the onset of metamorphosis), which appears from the larval metasomal sac and is divided into three parts (Fig. 8a). Each part has a unique organization of the epidermis and musculature, which is readily observed by CLSM (Fig. 8a). During metamorphosis, the two lateral parts of the larval preoral lobe are retained and give rise to the juvenile epistome (Fig. 5e). The epistome can be easily observed by CLSM in 4-day-old juveniles (Fig. 8b). Although most of remarkable external changes are completed, the newly formed juvenile retains a remnant of larval trunk, which looks like huge bulge on the anal side of the body. This bulge gradually undergoes reduction and completely disappears by day 9 after the onset of metamorphosis (Fig. 4d).

### Remodeling of the body cavities

**Fig. 9:**
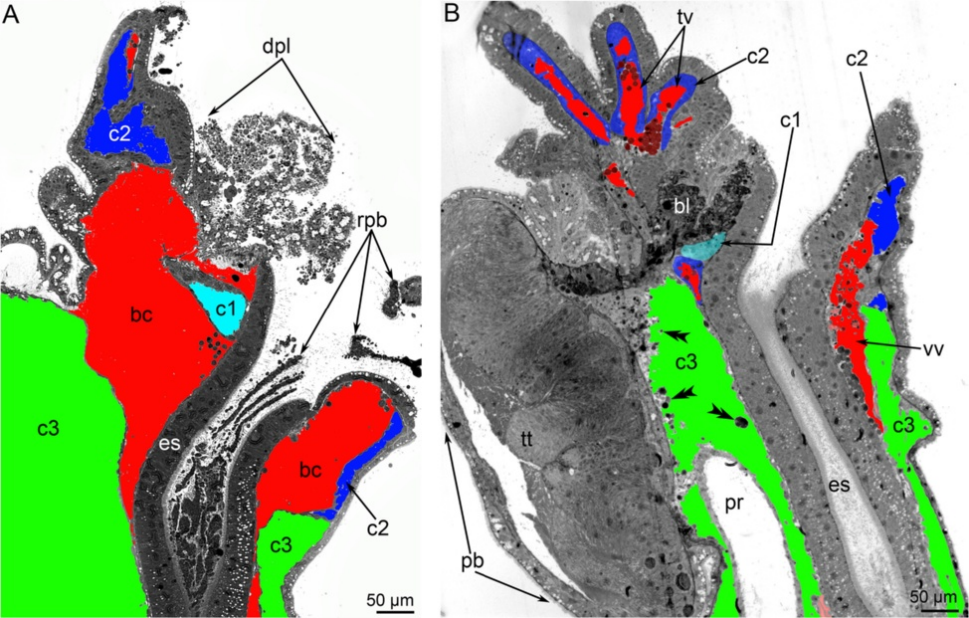
Metamorphic remodeling of the body cavities in anterior part of the body in *Phoronopsis harmeri*. Histological sagittal sections. The apical is to the top; the oral side is at the right; the anal side is at the left. Color code: red – blastocoel, which partly gives rise to the blood system; cyan – protocoel; blue – mesocoel; green – metacoel. **a** Metamorphic animal at stage of eating of the preoral lobe and the postoral ciliated band: about 10–15 min after the onset of metamorphosis. The degenerated preoral lobe (dpl) and ropes of the postoral ciliated (rpb) band are visible in the section. **b** 4-h-old juvenile with completely obliterated blastocoel and thick basal lamina (bl) instead of it. The lining of the posterior portion of the larval metacoel, which separate from the lamina and float in the metacoel, are indicated by double arrowheads. Abbreviations: bc – blastocoel; c1 – protocoel; c2 – mesocoel; c3 – metacoel; es – esophagus; pb – posterior bulge; pr – proctodaeum; tt – telotroch; vv – ventral vessel

The observations of the sagittal sections of animals at different stages of metamorphosis demonstrate that the volume of the blastocoel and protocoel decreases, whereas the volume of the meso- and metacoel does not decrease and may even increase (Fig. 4a–d). The blastocoel, which occupies the preoral lobe in larvae, is greatly reduced during metamorphosis because a portion of the preoral lobe is engulfed. The central portion of the preoral lobe, which contains the protocoel, remains (Fig. 9a), but its blastocoel disappears (Fig. 9b). Early in metamorphosis, the disappearance of the blastocoel is correlated with the formation of a thick layer of basal lamina, which contains thick electron-dense fibers and rests on fibrous ECM (Fig. 7b). The basal lamina forms very long projections, which penetrate into the ECM (Fig. 7c).

**Fig 10:**
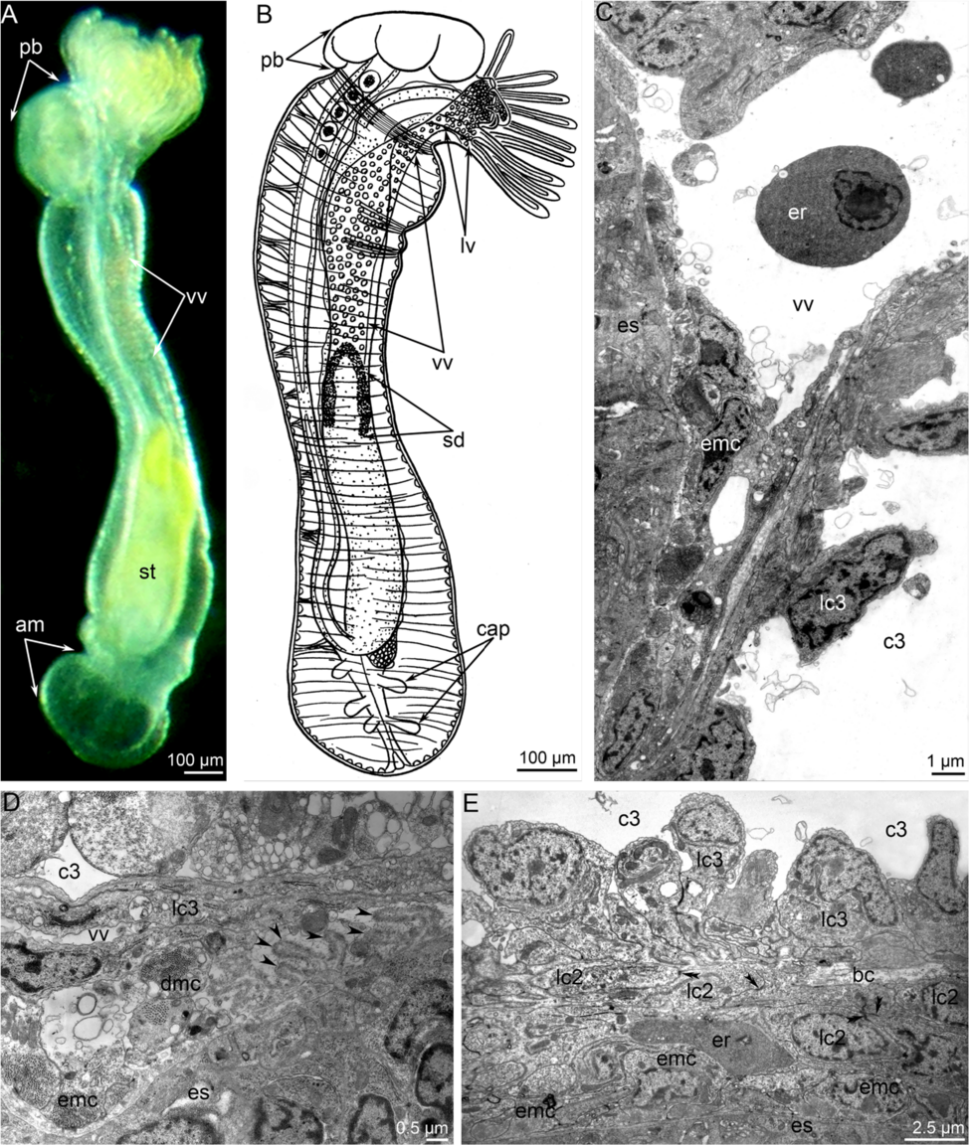
Metamorphic remodeling of the circulatory system in *Phoronopsis harmeri*. **a** Photographs of live 4-h-old juvenile with formed ventral blood vessel (vv). **b** Picture of 4-h-old juvenile with lophophoral (lv) and ventral blood vessels. **c** Ultrastructural organization of the ventral blood vessel. Cells (emc), which form muscular lining of the esophagus (es), form the inner lining of the ventral blood vessel. **d** Degeneration of muscle cells (dmc), which form the inner lining of the ventral blood vessel; the formation of long protrusions (are shown by arrowheads) of the basal lamina (bl). **e** The median blood vessel has a very complex wall along the upper portion of the esophagus. The wall is formed by lining of the metacoel (lc3) and two layers of the mesocoel lining (lc2). The muscle cells (emc) of the esophagus (es) form inner lining of the vessel. Desmosomes between cells of mesocoel lining are indicated by double arrowheads. Abbreviations: am – ampulla; cap – blood capillaries; c3 – metacoel; er – erythrocytes; pb – posterior bulge; sd – stomach diverticulum

The blastocoel of the collar region is greatly reduced during metamorphosis (Figs. 4b, c and 9a, b). It gives rise to the definitive lophophoral vessels, which initially form as one large circular vessel around the esophagus (Fig. 9a). This vessel extends into a spacious ventral blood vessel, which appears between the larval esophagus and metacoel lining (Fig. 10a, b). This lining is represented by the upper border of the metacoel, which does not contact the mesocoel. Because it is attached to the stomach diverticulum, this portion of the coelomic border is drawn into the everted metasomal sac during the first step of metamorphosis. The space between this border of the metacoel and the esophagus gives rise to the large ventral vessel. The larval esophagus stretches greatly and gives rise to the definitive prestomach (for details see [15]). Because the larval esophagus has its own muscular lining, which is formed by transversal and longitudinal muscles, the ventral blood vessel has an internal lining at the first stage of metamorphosis (Fig. 9c). Then muscle cells, which line the ventral side of the larval esophagus, die and degenerate; the cast-off cells are visible in the vessel (Fig. 10d). The basal lamina of the esophageal epithelium forms long protrusions, which extend into the vessel (Fig. 10d). Erythrocytes, which are released from the blood masses, are collected in the most spacious part of the ventral blood vessel, i.e., at the border between the larval esophagus and stomach diverticulum (Additional file 2). Because of strong movements of the body, digestive tube, and coelomic fluid, erythrocytes are pumped under the splanchnopleura of the stomach where blind capillaries develop as pouches of the larval ventral mesentery (Fig. 10b). During late stages of metamorphosis (stage about 20 min after the onset of metamorphosis), erythrocytes pass into the median blood vessel, which develops from the dorsal vessel of the larva. Along the stomach of the juvenile, the wall of the median blood vessel has the same fine structure as the larval dorsal vessel (for details see [28–31]), whereas the median blood vessel has a very complex wall along the upper portion of the esophagus (Fig. 10e). The muscular lining of the dorsal side of the esophagus is retained and forms the inner lining of the median vessel. The outer wall of the median vessel consists of an external layer, which is formed by the lining of the metacoel, and two internal layers, which are composed of the mesocoel lining (Fig. 10e). The juvenile (stage about 40 min after the onset of metamorphosis) has only one lophophoral, one ventral, and one median blood vessel. Blood capillaries already exist in tentacles of the larva; in the juvenile, the capillaries are filled with erythrocytes (Figs. 2d and 6g). In 4-day-old juveniles, the blood system acquires definitive structure and consists of two lophophoral, one lateroventral, and one median vessels (Fig. 2e).

*P. harmeri* larvae have a spacious blastocoel in the trunk between the body wall and the lining of the metacoel (Fig. 4a). During metamorphosis, this portion of the blastocoel undergoes reduction because of degeneration of the larval trunk, which becomes very short and forms a sack around the larval telotroch (Fig. 9b).

**Fig. 11:**
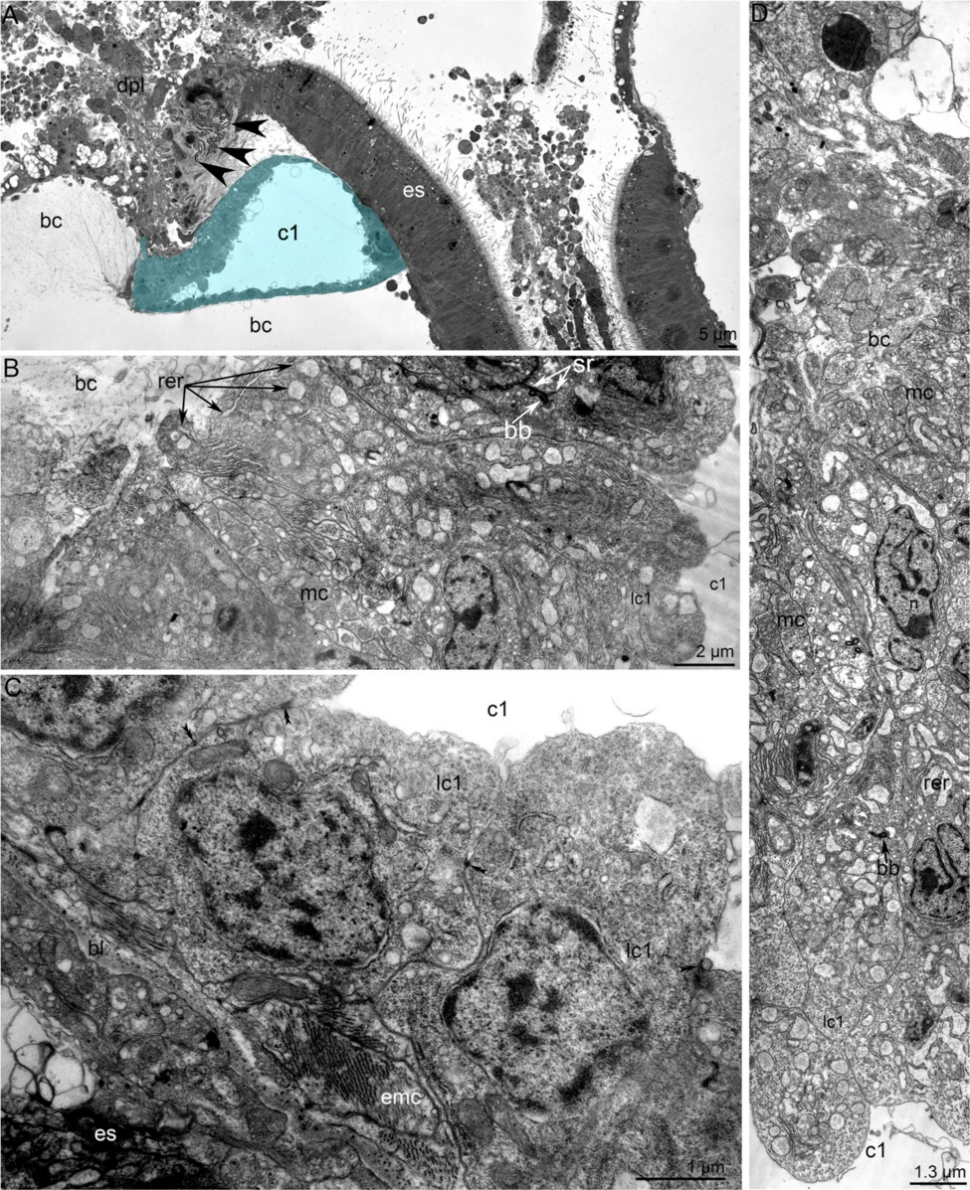
Organization of the protocoel at first stage of metamorphosis of *Phoronopsis harmeri*. **a** Sagittal semithin section of the protocoel (c1). Degenerated epithelium of the preoral lobe (dpl) is above the protocoel. Thick protrusions of basal lamina are indicated by arrowheads. **b** Thin section of the protocoel lateral wall, which includes large bundle of muscle cells. **c** Thin section of the protocoel lower wall. It contacts the esophagus (es) and muscle cells, which form the musculature (emc) of the esophagus. Desmosomes between cells of the protocoel lining are shown by double arrowheads. **d** The protocoel upper wall is formed by myoepithelial cells. Abbreviations: bb – basal body; bc – blastocoel; bl – basal lamina; lc1 – lining of protocoel; mc – muscular basal protrusions of cells of protocoel lining; rer – rough endoplasmic reticulum; sr – striated rootlet

**Fig. 12:**
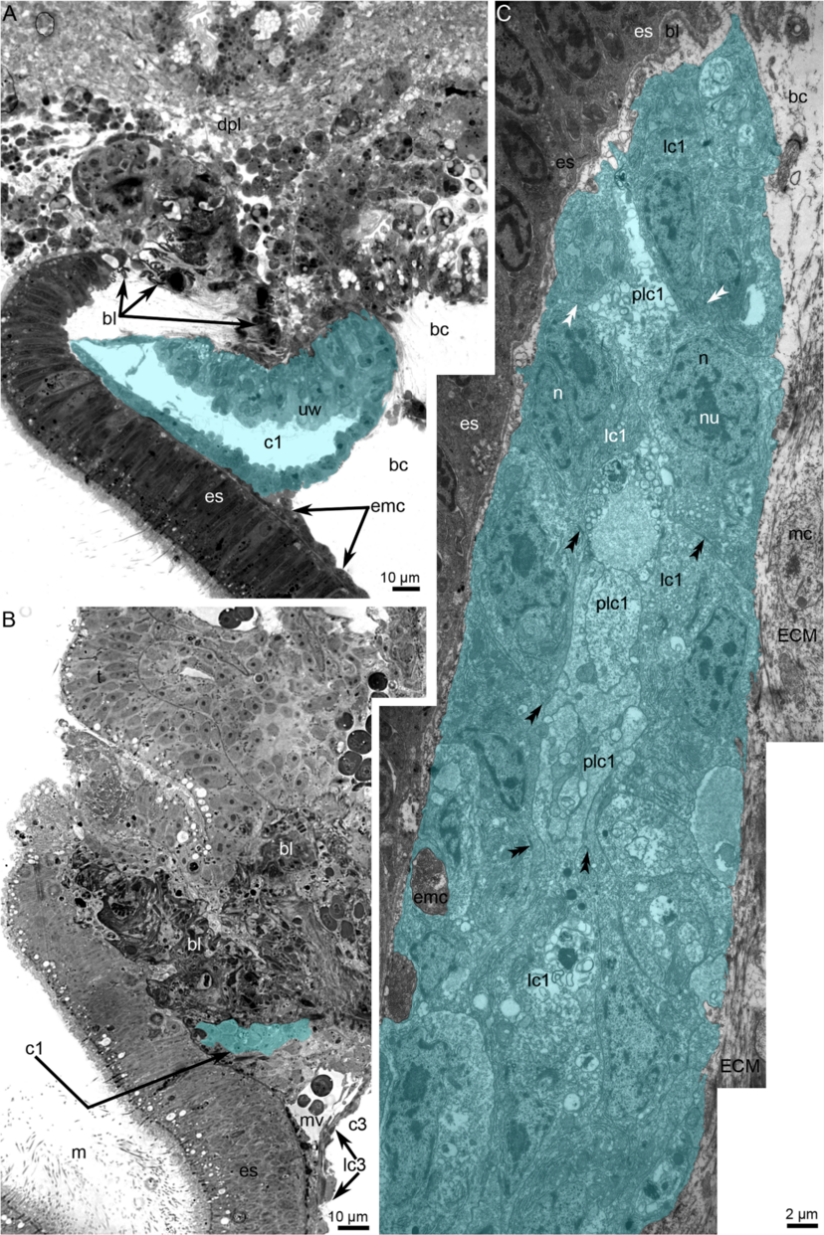
Organization of the protocoel at later stages of metamorphosis of *Phoronopsis harmeri*. **a** Sagittal semithin section of the protocoel (c1) at stage of eating of the peoral lobe. Degenerated epithelium of the preoral lobe (dpl) is above the protocoel. **b** Sagittal semithin section of the protocoel in 1-h-old juvenile. **c** Thin section of the protocoel of 1-h-old juvenile. The lumen of protocoel is filled with thick apical protrusions (plc1) of cells of the protocoel lining (lc1). Desmosomes between cells are indicated by double arrowheads. Abbreviations: bc – blastocoel; bl – basal lamina; c3 – trunk coelom; ECM – extracellular matrix; emc – muscle cells, which form esophageal musculature; es – esophagus; m – mouth; mc – muscle cells in blastocoel; n – nucleus; nu – nucleolus; mv – median blood vessel; rer – rough endoplasmic reticulum; sr – striated rootlet

During the first stages of metamorphosis (stages about 10–12 min after the onset of metamorphosis), the coelom in the metamorphic animal has the same organization as the coelom in the competent larva. The ratio of the volume of the blastocoel to that of the coelomic cavities also remains the same (Fig. 4a). There are no morphological or ultrastructural changes of the protocoel during first stages of metamorphosis. In early metamorphic stages, the volume of protocoel is large (Figs. 11a and 12a). The ultrastructure of its walls is similar to that in larvae [32]. The lateral walls contain large bundles of muscles, which are parts of the larval hood depressors [26]. The epithelial cells of the lateral walls are filled with mitochondria and rough endoplasmic reticulum (Fig. 11b). The lumens of the reticula are wide and contain electron-lucent material. Each cell has one cilium. The upper wall of the protocoel contacts the apical organ, which undergoes degeneration (Fig. 12a). The epithelium of the upper wall is formed by myoepithelial cells. Myofilaments are located in the basal portions of the cells, which usually form several thick projections (Fig. 11d). The nucleus has an irregular shape and contains a nucleolus. As in cells of the lateral walls, the cytoplasm of cells of the upper wall is filled with numerous mitochondria and rough endoplasmic reticulum (Fig. 11d). The upper wall of the protocoel is involved in apoptosis. Some cells of the upper wall of the protocoel closely contact the thick invaginations of the basal lamina, which appear in the sites of apoptosis (Fig. 7b). These invaginations start from the body wall, penetrate into the ECM, and branch many times, forming a complex net (Fig. 7c). The lower wall of the protocoel contacts the esophagus and consists of epithelial cells with electron-dense cytoplasm and a central, large nucleus (Fig. 11c). Muscle cells are located between the epithelial cells of the protocoel lining and the esophageal epithelium. These cells are inherited from the larva and compose the musculature of the esophagus. During the first stages of metamorphosis (stages about 10–12 min after the onset of metamorphosis), all cells of the protocoel are connected via desmosomes and are underlain by a basal lamina (Fig. 11c).

During the later stages of metamorphosis (stages about 20–25 min after the onset of metamorphosis), the protocoel appears as a small sack with a small lumen (Fig. 12b, c). The lumen of the protocoel is filled with thick, apical protrusions of cells of the protocoel lining. All cells of the protocoel have a similar ultrastructure. These cubical cells have a large nucleus with a nucleolus, numerous canals of rough reticulum, mitochondria, Golgi apparatus, and vesicles (Fig. 12c). Desmosomes occur between some cells but not others. The protocoel is surrounded by an extracellular matrix, but the basal lamina that underlies the cells of the coelomic lining is absent (Fig. 12c).

**Fig. 13:**
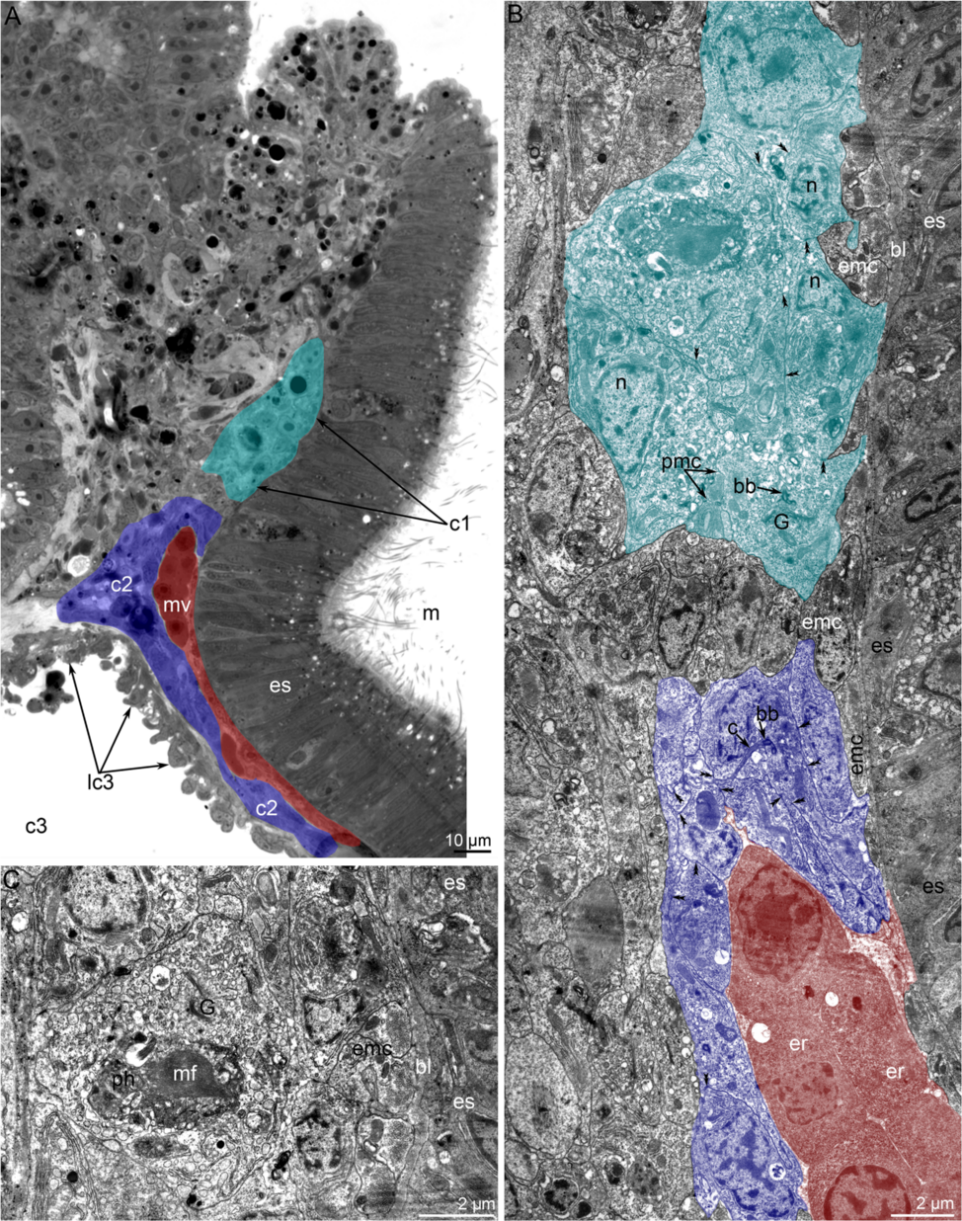
Organization of the protocoel in 4-day-old juvenile of *Phoronopsis harmeri*. Color code: red – median blood vessel; cyan – protocoel; blue – mesocoel. **a** Sagittal semithin section of the epistome. **b** Sagittal thin section of the protocoel (c1), mesocoel (c2), and median blood vessel (mv). Desmosomes are indicated by double arrowheads. **c** Portion of the protocoel lining (lc1). Large cell with phagosome (ph), which contains degenerated myofilaments (mf). Abbreviations: bb – basal body; bl – basal lamina; c – cilium; c3 – trunk coelom; emc – muscle cells, which form musculature of esophagus; er – erythrocyte; es – esophagus; G – Golgi apparatus; lc3 – lining of trunk coelom; m – mouth; n – nucleus; pmc – projections of muscle cells

In 4-day-old juveniles, the protocoel is very small (Fig. 13a, b). It almost lacks a lumen, which consists of a narrow space between the cells of the lining. These cells have a cilium and a large nucleus and are connected via desmosomes. The cytoplasm is filled with many vesicles, Golgi apparatus, rough endoplasmic reticulum, and mitochondria. Projections of muscle cells are infrequently embedded between cells of the coelomic lining (Fig. 13b). Some cells contain large membranous inclusions, which probably result from cell death and phagocytosis (Fig. 13c). These inclusions contain some fibrils, which are probably muscular. Muscle cells forming the circular musculature of the esophagus rest on the thick basal lamina of the esophageal epithelium (Fig. 13c).

**Fig. 14:**
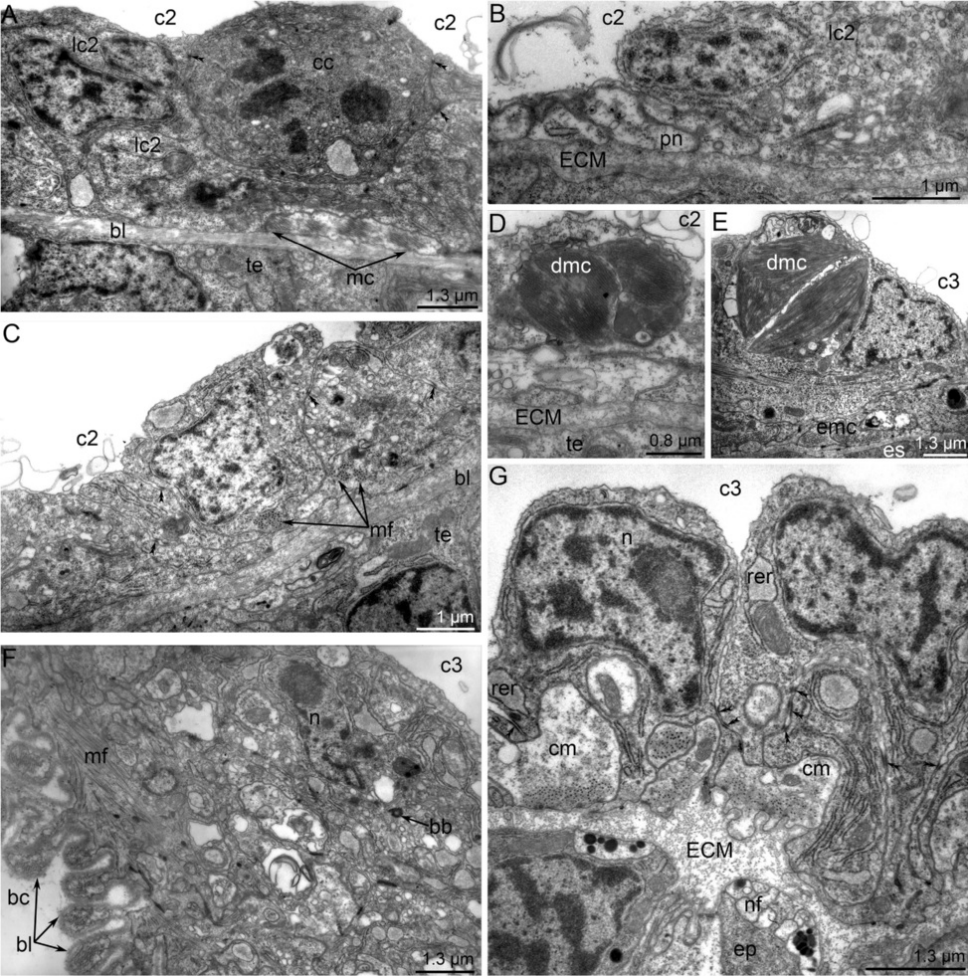
Ultrastructural details of the lining of meso- and metacoel during metamorphosis of *Phoronopsis harmeri*. Sagittal thin sections. First stage of metamorphosis (A, F); juvenile in 15 min after metamorphosis start (B); 1-h-old juvenile (D); 4-day-old juvenile (C, E, G). **a** Proliferating cell (cc) of coelomic lining. Well developed larval muscles (mc) are retained. **b** Presence of subperitoneal neurites (pn), which contain dense-core synaptic vesicles, under the mesocoel lining (lc2). **c** Completely established mesocoel lining. **d** Degenerated muscle cells (dnc) within cells of the mesocoel lining. **e** Degenerated muscle cells (dnc) within cells of the metacoel lining. **f** Metacoel lining: myoepithelial cells form long basal projections, which are covered by thick basal lamina (bl). **g** Somatopleura of the juvenile consists of myoepithelial cells, which form circular musculature (cm). Abbreviations: bc – blastocoel; bb – basal body; c2 – mesocoel; c3 – metacoel; ECM – extracellular matrix; emc – muscle cells, which form musculature of esophagus; mf – myofilaments; rer – rough endoplasmic reticulum; te – epidermis of tentacle

The mesocoel does not change greatly during metamorphosis. During the first stages of metamorphosis (stage about 10–12 min after the onset of metamorphosis), the mesocoel lining includes many cells, which proliferate (Fig. 14a). Cross-striated muscle cells that form larval tentacle elevators are visible at the base of the coelomic lining. During the later stages of metamorphosis (stages about 20–25 min after the onset of metamorphosis), subperitoneal nerve fibers are evident within cells of the coelom lining (Fig. 14b). At the end of metamorphosis (stage about 40 min after the onset of metamorphosis), peritoneal cells acquire their definitive structure: these are myoepithelial cells that are connected via desmosomes (Fig. 14c). Larval muscles undergo reduction: their degenerated parts are visible within the coelom lining (Fig. 14d).

The posterior portion of the larval metacoel undergoes reduction: its lumen disappears (Fig. 4a, d). The lining of the posterior portion of the larval metacoel is formed by pseudostratified epithelium. It consists of myoepithelial cells that form numerous basal projections, which are covered by a thick layer of the basal lamina (Fig. 14f). In 4-h-old juvenile, these cells separate from the lamina and float in the metacoel (Fig. 9b). Some of these cells are captured and apparently undergo phagocytosis by other cells of the metacoel lining (Fig. 14e). The new lining of the metacoel consists of myoepithelial cells, which have basal pouches containing the myofilaments (Fig. 14g). These pouches form a thin muscular net of the body wall (Fig. 8a).

## Discussion

### Larval morphology

The phoronid larva, i.e., the actinotroch, is a remarkable one. It is the only type of primary invertebrate larva with a blood system. In phoronids, the larval blood system consists of a spacious blastocoel, which is located in the collar region and contains several masses of erythrocytes, and several blood vessels extending along the digestive tract [28]. The larval blood system does not function but is completely formed. Such an unusual feature, i.e., the presence of a blood system in the larval stage, can be regarded as a developmental acceleration, which correlates with the presence of a very complex blood system in adults [29–31]. Another example of such an acceleration is the organization of the larval protonephridia, which have a U-shaped duct in *Phoronopsis harmeri* larvae [33]. In other phoronids, there are the nephridia that become U-shaped during metamorphosis [34].

In phoronid larvae, the coelom is organized in two different ways [32]. Larvae of *Phoronopsis* spp. have a closed coelomic cylinder in the preoral lobe under the apical organ, whereas larvae of *Phoronis* spp. lack this coelomic cylinder [20]. In the latter case, larvae have only a dorsal septum that extends between the dorsal border of the apical organ and the esophagus. This septum is formed by epithelial cells, which are connected via desmosomes (unpublished data).

### Remodeling of external morphology

Phoronid metamorphosis is an unusual and rapid process: most of the external remodeling occurs within several minutes [17, 35, 36]. Phoronida is regarded as a group, whose metamorphosis is one of the fastest among all animals with pelagic larvae [1, 12]. Fast metamorphosis allows animals start to feed very soon after larval settlement [12]. Phoronids start to eat immediately after metamorphosis beginning, because they first consume huge part of larval body. The same process of eating of whole larval body was recently described in nemerteans [37].

As recently demonstrated, phoronid metamorphosis occurs in one of two ways [15, 26, 27]. During metamorphosis, most phoronids studied to date pull the larval telotroch into the proctodaeum, where the telotroch undergoes digestion [23, 35, 36, 38, 39] (Table 1). *P. harmeri*, in contrast, retains the larval trunk and the telotroch for 9 days; these larval structures undergo a slow cell death and then disappear [15].

**Table 1:** Fate of some larval structures during metamorphosis in studied phoronid species

Name of studied species and author of study	Fate of larval organs			
	Preoral lobe	Tentacles	Telotroch	Protocoel
“Actinotrocha A” (probably larva of *Phoronopsis harmeri*) [22]	transforms into small unpaired remnant that gives rise to the adult epistome	completely turns into definitive tentacle	retained for at least 4 days and then degenerates	**?** “…the existence of a coelomic epithelium in the juvenile epistome is impossible confirm or deny…”
*Phoronopsis harmeri* [15, 26, 27, herein]	is partly retained as two lateral remnants, which contribute to the future formation of the epistome	undergo partial reduction: the postroral ciliated band degenerates. Larval tentacles change neither in length nor number	retained for 9 days and then disappears	retained, but greatly reduces in size
*Phoronis muelleri* [23, 35]	retained as fold of the larval episphaere near the mouth and then turns into epistome	completely destroyed; adult tentacles arise from anlagen of definitive tentacles, which are located under the larval	is drawn into the digestive tract and becomes hindgut	cells, which form the lining of the preoral lobe are completely retained and give rise to the definitive protocoel
*Phoronis muelleri* [39]	**?**	completely destroyed; adult tentacles arise from anlagen of definitive tentacles, which are located under the larval	is drawn into the digestive tract and becomes hindgut	protocoel is present neither in larva nor adult
*Phoronis psammophilla* [24]	retained as fold of the larval episphaere near the mouth and then turns into epistome	completely destroyed; adult tentacles arise from anlagen of definitive tentacles, which are located under the larval	retained for several days and then disappears	**?**
*Phoronis pallida* [25]	completely destroyed	undergo great cellular death. Accordingly to figures, the length and number of larval tentacle greatly reduce. Definitive tentacles apparently arise from proximal portions of larval tentacles.	**?** can not be observed by SEM in juvenile just after the completion of metamorphosis. Apparently, the telotroch is drawn into the digestive tract	**?**
“Actinotrocha C” [38]	completely destroyed	thickened proximal portions are retained through metamorphosis and give rise to definitive tentacles	is drawn into the digestive tract	**?**

“**?**” – the fate of larval organs unknown or not described in detail

The fate of larval tentacles also differs among phoronids. Larvae of some phoronids have definitive tentacles under the larval tentacles [13, 16, 17, 40]. In these larvae, the definitive tentacles give rise to the adult tentacles, and the larval tentacles are completely engulfed early in metamorphosis. Another mode of tentacle remodeling occurs in *P. harmeri* in that the larval tentacles become the adult tentacles. The exception in *P. harmeri* is the larval postoral ciliate band, which undergoes cell death. Thus, the definitive postoral ciliated band arises *de novo* in *P. harmeri* (Table 1). The postoral ciliated band is the most important structure for food capture, which occurs in different ways in the larva and the adult [41]. Remodeling of the larval postoral ciliated band in *P. harmeri* correlates with the change in how food is captured by the adult. At the same time, *P. harmeri* demonstrates the minimal remodeling of larval tentacles: they change neither in length nor in number. Moreover, muscles of larval tentacles are partly retained and integrated into definitive muscles [26]. These minimal changes of larval tentacles allow the juvenile to use tentacles for food capture in little time after catastrophic remodeling. Phoronids, whose larval tentacles are completely consumed by the juvenile, have to raise definitive tentacles in some time. During this period, the juvenile digests larval preoral lobe, tentacles, and telotroch [13].

The fate of the larval preoral lobe (= the hood) and the formation of the adult epistome are unclear during phoronid metamorphosis. There are two main views about the fate of the larval preoral lobe. The first view, which has been discussed in many early papers [42–48], supposes that the larval preoral lobe undergoes total reduction, whereas the adult epistome arises *de novo*. The second view was first suggested by Siewing [23], who studied the metamorphosis of *Phoronis muelleri* and discovered that a small part of the larval preoral lobe is retained and gives rise to the adult epistome. This second view has been supported by many studies [27, 49–51]. According to our results, *P. harmeri* retains two dorso-lateral portions of the larval hood, which together with the larval esophagus form the adult epistome. Thus, in *P. harmeri*, the central portion of the epistome arises from the dorsal wall of the larval esophagus, whereas the lateral parts of the epistome originate from the larval hood.

Table 1 demonstrates that there are two different descriptions, based on different studies for two phoronid species: *P. harmeri* [15, 22, 26, 27] and *Phoronis muelleri* [23, 35, 39]. This difference of descriptions might be explained by the use of different methods: only light microscopy for early studies [22, 23, 35] and electron and laser confocal microscopy for recent researches [15, 26, 27, 39]. The use of modern methods allows to recover new details, which were unknown before.

### Remodeling of the body cavities

The formation of the definitive blood system was described in detail in some early papers [44, 52]. These authors mentioned that the cavity of the larval collar gives rise to the adult lophophoral vessel. Both Ikeda and Cowles regarded the collar cavity of phoronid larvae as a coelom. For this reason, they could not explain how the connection was established between the lophophoral and longitudinal vessels. Our previous results [28] and current results clearly show that the larval collar cavity is the blastocoel, which connects the larval vessels that are slits between the wall of the larval digestive tract and the lining of the metacoel. The formation of the ventral blood vessel, which arises between the larval esophagus and the upper border of the metacoel, was described by Cowles [52], whose findings are supported by our results. The dorsal vessel of the larva gives rise to the median vessel of the adult. The future development of the definitive blood system correlates with the formation of the septum in the lophophoral vessel and its division into upper and lower portions (afferent and efferent lophophoral vessels) as well as with the formation of two lateral blood vessels, which are descendants of the ventral blood vessel.

The most discussed problem of phoronid metamorphosis is the fate of the preoral coelom. Most researchers have inferred that the definitive preoral coelom develops from the proximal portion of the larval protocoel, which is located at the posterior border of the apical organ [22–24, 51]. According to these authors, the larval protocoel occupies all of the volume of the larval preoral lobe, from its edge to the dorsal septum. Exactly this septum is regarded as anlage of the definitive preoral coelom [51]. As recently shown [39], some phoronid larvae lack a protocoel. These phoronid species probably lack a protocoel as adults [53]. Interestingly, *P. harmeri* has a protocoel in larval stages [32] and in adults [54]. Nevertheless, the protocoel greatly decreases in size during metamorphosis and partly loses its epithelial integrity: late in metamorphosis, some cells of the protocoel lack desmosomes. This may reflect the reduction of the protocoel, which occurs in many other phoronids [39, 53] and perhaps occurred in phoronid evolution [55].

### Phylogenetic implication

The phylum Phoronida has been classified into the protostomian clade through molecular phylogenetic analyses [56, 57]. However, phoronid morphology and embryology have more in common with deuterostomes than protostomes [58, 59]. For this reason, the affiliation of phoronids with the protostomian clade cannot be regarded as strictly established. Phoronids have been traditionally considered as a group within clade Lophophorata [14, 60–62], whose unity was strongly criticized [63, 64], but recently is restored by molecular [65, 66] and morphological data [67, 68]. All adult lophophorates have tentacles, which are used for food capture, brooding, respiratory, and as a sensory organ [14]. Most of phoronids have planktotrophic larvae, which live in plankton for several weeks and use tentacles for capture of food particles [13, 16, 17]. According to our data, *P. harmeri*, which demonstrates numerous plesiomorphic features in organization of different organ systems [54, 69], have tentacles as larvae, juvenile, and adult. The plesiomorphic nature of *Phoronopsis* together with its retention of larval tentacular musculature [26] and its direct transformation of larval tentacles into juvenile tentacles suggests that the presence of tentacles is a primary phoronid characteristic, i.e., the phoronid ancestor had tentacles at larval and adult stages. Among brachiopods, which are the closest relatives of phoronids [70, 71], the most primitive species of genus *Lingula* have planktotrophic larvae, whose tentacles directly transform into the juvenile tentacles. This fact might be regarded as an additional evidence for the presence of tentacles in the last common ancestor of clade Brachiozoa (Phoronida + Brachiopoda).

On the other hand, among phoronids there is only a species with lecitotrophic larvae – *Phoronis ovalis* [13]. This tiny phoronid is characterized by the production of a few large eggs, which are bred in the mother’s tube. *P. ovalis* is regarded as the most primitive species among phoronids [70, 72]. In the light of the basal position of *P. ovalis*, the primacy of lecitotrophic larvae in phoronids seems to be more plausible than the primacy of planktotrophic larvae. In this case, we have to suggest that the phoronid ancestor did not have tentacles and tentacles appeared first in small phoronids in a small number and then increased in number in large phoronids.

Thus, we can see two possibilities for the evolution scenario of the last common phoronid ancestor. If we accept the first scenario, we have to suppose the reduction of larvae with tentacles in some phoronids, most of brachiopods, and all bryozoans. In contrast, if we agreed with second scenario, we have to imagine independent origin of tentacles in all three groups of the lophophorates. In the light of the available evidence, the first scenario seems more plausible than second scenario.

The organization of the coelomic system is traditionally used for the establishment of relationships between large taxa. The presence of three coelomic compartments in phoronid larvae and adults, has been traditionally regarded as an important evidence of close relations between phoronids (and other lophophorates) and deuterostomians. Among lophophorates, three coelomic compartments are discovered in some phoronid larvae [32, 51], in several adult phoronids [54, 55], and in only an adult brachiopod – *Lingula anatina* [73]. Because phoronids and brachiopods have unusual lateral mesenteries in the trunk coelom, we have recently suggested an idea about the metameric organization of these animals [21]. According to this idea, the last common ancestor of the lophophorates had protocoel, mesocoel, and metacoel. The protocoel and metacoel are specialized coelomic compartments, which occupy the two first segments of the body. The metacoel occupies the trunk and consists of two segments in phoronids and three segments in brachiopods. These segments of trunk are divided by lateral mesenteries. In the light of this idea, the presence of the coelom and segmentation seem to be plesiomorphic condition for the lophophorates, as it likely is for the last common bilaterian ancestor [21]. This idea is completely corroborated with numerous data that the last common ancestor of Deuterostomia, Ecdysozoa and Spiralia had a segmented and coelomate body organization and that morphologically more simply organized taxa evolved by secondary reductions [74–78]. On the other hand, our previous idea is in stark contrast to the traditional “acoeloid–planuloid” hypothesis favoring evolution of Bilateria from a simple body organization toward more complex forms with a last common ancestor resembling a flatworm without segmentation and coelomic cavities [60, 79–82].

Because all lophophorates have tentacles and coelom, which usually is subdivided into several compartments, we can conclude that the last common ancestor of the lophophorates was a coelomic animal with tentacles. Whether these structures were inherited from the last common bilaterian ancestor or acquired independently – this question demands further researches and analyses.

### Evolution of the phoronid body plan

To date, the only idea about the formation of the body plan in recent phoronids was suggested by Mamkaev [19]. He supposed that a worm-like phoronid ancestor lived in a U-shaped burrow in soft sediment. Given the shape of the burrow, the body of the worm was also U-shaped. With time, two parts of this U-shaped body of the phoronid ancestor gradually approached each other and finally merged. The lateral mesenteries, which are unique to adult phoronids [13], appear as a result of this merging. It is important to consider that Mamakev did not study phoronid metamorphosis; his interpretation was based solely on the observation of adult phoronids [19].

**Fig. 15:**
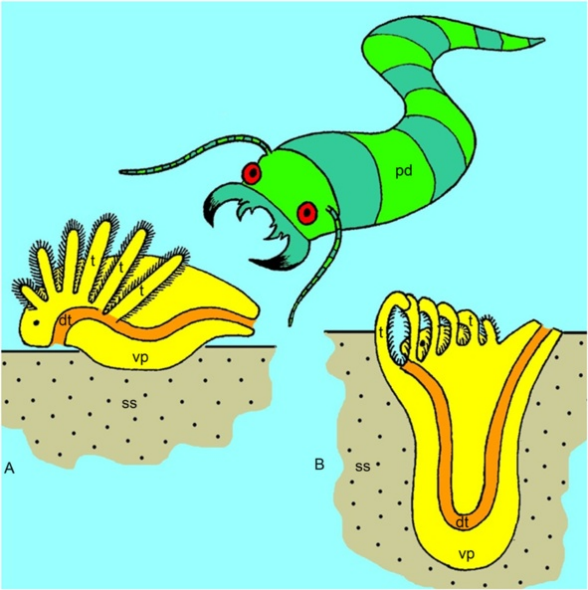
Hypothetical scenario of evolutionary formation of the phoronid body plan. **a** The last common phoronid ancestor, which was worm-like animal, had tentacles for food capture, and moved on the ventral side of the body. **b** Had being in danger, the worm-like ancestor buried itself in soft sediment by means of the ventral protrusion, to which the loop of the intestine was drawn. Abbreviations: dt – digestive tract; pr – hypothetical predator; ss – soft sediment; t – tentacle; vp – ventral protrusion

Our investigation of phoronid metamorphosis indicates that the phoronid larva does not acquire a U-like shape and does not fold in two. The most prominent feature of phoronid metamorphosis is the eversion of the metasomal sac, which is a ventral pouch of the body. The terminal portion of this pouch is the ampulla, which is quite flexible and can repeatedly increase and decrease in diameter. We hypothesize that the phoronid ancestor was a worm-like animal that lived on soft sediment and used tentacles for food capture (Fig. 15a). Like many other invertebrates, this ancestor used its ventral side for movement. When this worm-like phoronid ancestor was threatened by predators, it used its muscular ventral surface to dig into the soft substratum (Fig. 15b). We propose that this pattern of behavior resulted in the evolution of a new body plan in recent phoronids and that this evolution is reflected in the metamorphosis of recent phoronids.

### Evolution of the phoronid life cycle

**Fig. 16:**
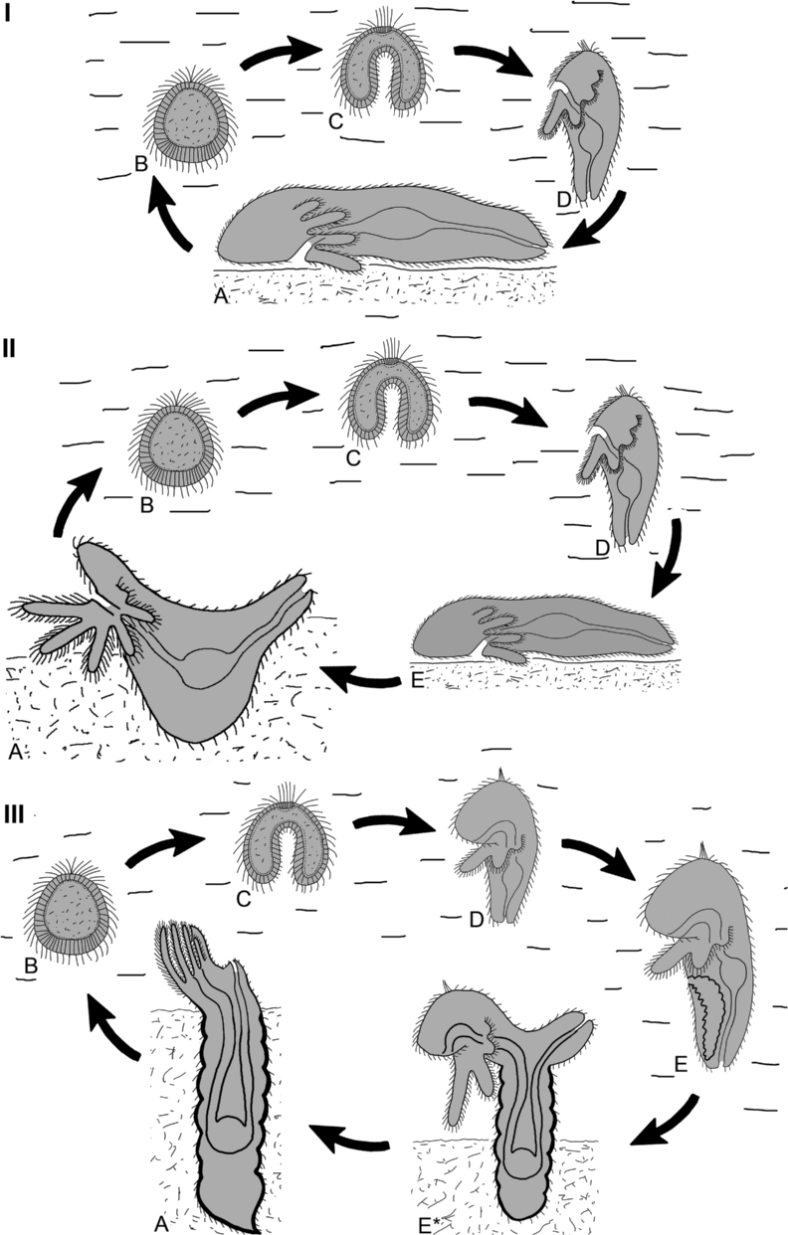
Hypothetical scenario of evolutionary formation of the phoronid life cycle. **I** Life cycle of the last common phoronid ancestor, which was a benthic animal and had planktotrophic larva (lophotrochophore). **II** Life cycle of hypothetical ancestor, which was a sessile animal with pelagic larva and benthic creeping juvenile. **III** A generalized scheme of the life cycle of recent phoronids, which have pelagic larva and pelagic competent larva that is a juvenile. Abbreviations: a – adult animal; b – blastula; c – gastrula; d – planktotrophic larva; e – pelagic juvenile; e* – settled juvenile

Recently, the origin and evolution of biphasic life cycle is actively discussed and two theories called “terminal addition theory” and “intercalation theory” were proposed for the explanation how pelagic larvae could arise [1–12]. Without reference to the way of primary origin of pelagic larvae in early metazoan evolution, there can be no doubt that the phoronid ancestor already had a biphasic life cycle inherited from ancient lophotrochozoan predecessors (Fig. 16I). The adult organism lived as benthic animal and moved at the surface of the substrate. The adult spawned eggs into the water. Eggs developed into a ciliated blastula, which floated in the water and then transformed into the gastrula. Gastrula gave rise to more complicated larva (lophotrochophore), which swam in the water for some time and then settled down. The larvae did not undergo any catastrophic changes, gradually acquired definitive features, and turned into the adult (Fig. 16I). The second step of the evolution of phoronid life cycle correlates with the appearance of the sessile stage and the formation of a new body plan (Fig. 16II). At this step the larva, which swam in the water, gave rise to the juvenile that moved on the substrate and then dug into it, trying to escape a predator attacks. The abilyty of juvenile to dig into the ubstratum was acquired at this step of the life cycle. It was not just event in the life of an individual, but an evolutionary step. The third step of phoronid life cycle evolution we can find in most of recent phoronids (Fig. 16III). At this step, the stage of ancestral juvenile, who moved on the substrate, is transformed into competent actinotrocha. As we mention before, competent phoronid larva has some specific features, which can be regarded as a juvenile’s peculiarities: the presence of blood system, fully developed canals of nephridia, and the metasomal sac. The competent larva undergoes the eversion of the metasomal sac and turns into the definitive animal. Thus, in phoronids, we can see the prolongation of the pelagic part of the life cycle due to intercalation of new stages, which may be regarded as juveniles adapted to pelagic mode of life. The evolution of phoronid life cycle in this way seems having more in common with“intercalation” than “terminal addition” theories.

## Conclusion

In this research, we used modern methods to provide a detailed description of phoronid metamorphosis. Our results reveal the metamorphic remodeling of some larval organs and body cavities, whose fates were previously unclear. We report that metamorphosis in *P. harmeri* differs from that in other phoronids studied to date. In *P. harmeri*, for example, remodeling of larval tentacles involves degradation of the postoral ciliated band. The preoral lobe of the larva is retained as two lateral remnants, which then contribute to the formation of the definitive epistome. The telotroch is not drawn into the digestive tract, as it is in most of other studied phoronids, and it gradually degenerates over 9 days (Table 1). Our report reveals that the *P. harmeri* protocoel is maintained during metamorphosis and gives rise to the definitive protocoel, which is well-developed in adults [54]. Our new data confirm previous assumption that the phoronid metamorphosis occur in two different ways: with complete and incomplete reduction of larval structures. *P. harmeri* demonstrates minimal changes of larval structures: in contrast to other studied phoronid species, it retains larval protocoel, tentacles, and digestive tract. Because the protocoel (as any other coelom) functions as a skeleton of the epistome, it is important to retain it in *P. harmeri*, which has a large movable epistome. Direct transformation of larval tentacles into the definitive tentacles allows the juvenile to capture food particles immediately after the critical point of the metamorphosis.

On the other hand, metamorphosis of all phronids demonstrates a general pattern common to all species studied to date. Phoronid metamorphosis starts from eversion of the deep invagination of larval ventral side of the body (a metasomal sac). The metasomal sac gives rise to the most part of adult body. The terminal portion of the metasomal sac (ampulla) demonstrates enormous mobility and is used by the juvenile for burying into the substrate. The presence of movable ampulla and large metasomal sac in all phoronids may reflect the significance of the ventral side of the body in phoronid ancestor. Many recent invertebrates use the ventral side of the body to escape predators by digging into substrata. For this reason, we have suggested an idea about evolutionary formation of the phoronid body plan due to inheritance of an acquired modus of escaping development.

## Additional files

Additional file 1:
**Real-time video of the typical 4-h-old juvenile of** ***Phoronopsis harmeri*** **.** The ampulla is quite flexible and can repeatedly increase and decrease in diameter. In the middle part of the body, the ventral blood vessel, which is filled with numerous red erythrocytes, is visible. (AVI 8216 kb) Additional file 2:
**Real-time video of typical movements of the ampulla in the 4-h-old juvenile of** ***Phoronopsis harmeri*** **.** Transparent integument allows to see peristaltic movements of the main blood vessels (at the left) as well as movements of numerous blind capillaries, which are developed on the oral-anal mesentery (former ventral mesentery of the larva). (AVI 14245 kb)
